# Controlling the Optical Properties of Gold Nanorods in One-Pot Syntheses

**DOI:** 10.1021/acs.jpcc.1c10447

**Published:** 2022-02-03

**Authors:** Lucien Roach, P. Louise Coletta, Kevin Critchley, Stephen D. Evans

**Affiliations:** † School of Physics and Astronomy, University of Leeds, Leeds LS2 9JT, U.K.; ‡ Leeds Institute for Medical Research, University of Leeds, Leeds LS2 9JT, U.K.

## Abstract

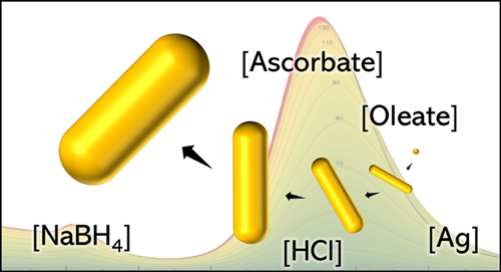

We present the characterization of the CTAB-oleate controlled synthesis of gold nanorods (AuNRs). Concentrations of key compounds in the synthetic system were varied in the presence of oleate, including HCl, borohydride, silver nitrate, and ascorbic acid. The longitudinal surface plasmon resonance peak was sensitive to changes in all concentrations. Reducing the concentration of Ag ions below 66 μM led to slower reaction kinetics and incomplete Au reduction. Variation of the ascorbic acid concentration revealed that oleate is responsible for around 44% of reduction of Au^3+^ to Au^+^ before nucleation in these experiments. Increasing the oleate concentration significantly slows the growth kinetics and leads to much longer synthesis times of above 12 h for reaction completion. These observations will enable the design of better methods of synthesizing of AuNRs using binary surfactants.

## 1 Introduction

Gold nanorods (AuNRs) are one of the most heavily researched gold nanoparticle morphologies.^1−4^ AuNRs have narrow extinction peaks tunable throughout red and near-infrared through the control of their aspect ratio and their dielectric environment.^5^ They also offer the largest plasmonic response per unit mass of all Au nanoparticles, capable of strong field enhancement at their tips and efficient conversion of light-to-heat.^6−8^ This combination of properties has meant that they have found applications in roles such as optical sensors,^9^ surface-enhanced Raman spectroscopy probes,^10^ and photothermal conversion agents in photothermal therapy and photoacoustic imaging.^1,11^ Thus, there is considerable interest in the synthesis of AuNRs and controlling their optical properties.

Jana et al. published the first seeded AuNR synthesis in 2001, which reduced a gold salt on to pre-synthesized Au seeds in a cetyltrimethylammonium bromide (CTAB) solution using ascorbic acid (AA).^11^ This protocol has since seen many modifications, of note are the inclusion of silver ions^12^ and the optimization of the reaction pH,^14^ which both resulted in an improved yield of rod-like nanoparticles. The inclusion of silver salts also notably resulted in the production of monocrystalline AuNRs (as opposed to pentatwinned AuNRs). More recent innovations have focused on the inclusion of additives in the growth solution to further improve the yield and monodispersity of the synthesized AuNRs. These have broadly consisted of (1) aromatic molecules, such as salicylate,^15,16^ dopamine,^17^ hydroquinone,^18^ or resveratrol;^19^ (2) co-surfactants like benzyldimethylhexadecylammonium chloride^13,20^ and oleate;^21−24^ or (3) Hoffmeister salts,^25^ as well as replacing CTAB with alternative surfactants such as dodecylethyldimethylammonium bromide^26^ or Gemini surfactants.^27^ These improvements have made AuNR syntheses more reliable, improved the quality of the end products, and provided additional means to control the optical properties (and hence the morphology) of synthesized AuNRs. AuNRs can also be synthesized through the direct reduction of HAuCl_4_ in the growth solution using a strong reducing agent such as NaBH_4_ without the presence of seeds in “one-pot” (or “seedless”) protocols, which can also produce monodispersed AuNRs of high purity.

However, there remains a debate on the exact processes that occur during the synthesis of AuNRs and the role of each component. For instance, the mechanism by which Ag ions function as a facet-specific capping agent is still debated. Both the underpotential deposition of a Ag^0^ monolayer^28^^–^^31^ and the formation of surface-bound CTA–Ag–Br^28,32^ have been suggested. Currently, the opinion in the literature mostly favors the former, but it has not been conclusively proven as the mechanism. The role of the reducing agent AA is still not fully understood. Some researchers suggest that AuNR growth occurs through an autocatalytic disproportionation reaction (3Au^+^ → 2Au^0^ + Au^3+^) with the AA reducing the produced Au^3+^ ions back to Au^+^ to continue the reaction.^33^ Whereas others suggest that it directly reduces Au^+^ on the surface of the AuNR.^34^ The latter has become more widely accepted following reports that no particle growth occurs when AA is replaced with weaker reducing agents, such as salicylate and oleate. These can reduce Au^3+^ to Au^+^ but not Au^+^ to Au^0^, meaning that these cannot drive particle growth in the absence of a disproportionation reaction and hence direct reduction is more likely as the growth mechanism.^15,21^

We previously reported that the aspect ratio of AuNRs was tunable through variation of the surfactant concentrations used in a “one-pot” binary surfactant-based protocol.^22^ The inclusion of a second surfactant, sodium oleate, improves the monodispersity and shape yield of the synthesized nanorods.^21,22^ However, the introduction of oleate alters the properties of the reaction medium and changes the reactant concentrations required for AuNR growth. Here, we report the effects of changing the concentrations of HCl, borohydride, silver nitrate, and AA on AuNR syntheses in the presence of oleate. We also have investigated the effect of oleate on the kinetics of the synthesis.

## 2 Methods

### 2.1 Materials

L-Ascorbic acid (A15613) was purchased from Alfa Aesar. Hydrochloric acid (A144S-500), silver nitrate (11414), and sodium borohydride (10599010) were purchased from Fisher Scientific. Gold (III) chloride trihydrate (520918) and cetyltrimethylammonium bromide (H6269) were purchased from Sigma-Aldrich. Sodium oleate (O0057) was purchased from TCI. All solutions were prepared using Milli-Q grade deionized water (18 MΩ cm).

### 2.2 Synthesis of Gold Nanorods

AuNRs were synthesized following our previously reported protocol.^23^ AuNRs were prepared in 10 mL batches. The volumes and concentrations were varied as part of this study, but in a typical synthesis, solutions of CTAB and sodium oleate (200 mM) were prepared in advance, by heating to 70 °C under stirring until all the solute was dissolved. The solutions were cooled to 30 °C before use. Vials were cleaned with aqua regia and thoroughly rinsed, before 2.4 mL of CTAB, 0.625 mL of oleate, and 1.925 mL of water were added and mixed. This was followed by the sequential addition 5 mL of HAuCl_4_ (1 mM), 240 μL of AgNO_3_ (4 mM), 50 μL of HCl (11.8 M), and 75 μL of AA (85.8 mM). To this 7.5 μL of freshly prepared ice-cold NaBH_4_ (10 mM) was rapidly injected into the mixture. The mixture was then held at 30 °C for 4 h. The AuNRs were isolated by centrifugation at 9000*g* for 30 min. The supernatant was discarded, and the precipitate was resuspended in water. AuNR solutions were stored in the dark at room temperature. The concentrations given here are examples, any deviations from this protocol are stated in the main text. All concentrations are given as the concentration in the total volume of the growth solution after the addition of all solutions (i.e. 2.4 mL of 200 mM CTAB in a 10.4 mL of growth solution yields [CTAB] = 46.3 mM).

### 2.3 UV–Vis Spectrometry

For individual spectra, measurements were taken using an Agilent Cary 5000 UV–vis–NIR using quartz cuvettes (*L*_path_ = 1 cm). Samples were typically diluted by a factor of 10 before spectra acquisition. Where spectra are presented un-normalized, they have been multiplied by the dilution factor to account for this (following the Beer–Lambert law).

For kinetic spectra, AuNR growth solutions were prepared as described above in a 10 mL vial, after the addition of the NaBH_4_, the solution was mixed quickly and 700 μL was pipetted into a quartz cuvette (*L*_path_ = 2 mm) and the capture of spectra immediately started. Cuvettes were cleaned with aqua regia and rinsed thoroughly before use. During the first 5 h, spectra were taken at 2 min intervals at 1800 nm min^–1^. For the following 24 h, spectra were taken at 10 min intervals at 900 nm min^–1^. Solutions were maintained to 30 °C throughout spectra acquisition. To account for the reduced path length, spectra from these measurements are multiplied by a factor of 5 to retrieve the true extinction value (again following the Beer–Lambert law).

Where concentrations have been calculated from the extinction at 400 nm (*A*_400nm_), this was done using the approximation [Au^0^] = *c*·*A*_400nm_ (where *c* = 0.42 mM) given elsewhere in the literature.^35−37^ This was confirmed experimentally using a Varian 240 fs atomic absorbance spectrometer (Figure S1). In syntheses, which vary the concentration of Ag ions, the presence of Ag^0^ is expected to have very little impact on *A*_400nm_, because the intraband transitions of Ag occur below this wavelength.^5^

### 2.4 Electron Microscopy

Transmission electron microscopy (TEM) images were obtained using a Tecnai G2 Spirit TWIN/BioTWIN with an acceleration voltage of 120 kV. TEM samples were prepared by drying ∼5 μL of 10× concentrated nanoparticle dispersion on an amorphous carbon-coated 400-mesh copper grid (Electron Microscopy Services, CF400-Cu).

## 3 Results

In a typical monocrystalline AuNR synthesis, a growth solution is prepared containing shape-directing surfactants (in this case CTAB and oleate), a gold salt, and a silver salt (typically ∼5:1 Au/Ag molar ratio). A small volume of HCl is then added to this, followed by a mild reducing agent, such as AA. HCl is included to lower the pH to prevent the nucleation of gold particles by AA. Finally, either a thermally aged seed solution is added, or NaBH_4_, a strong reducing agent which induces the nucleation of gold nanoparticles, is added. Further Au reduction then occurs onto these seeds with the surfactants and silver causing anisotropic growth. This study focuses solely on a “one-pot” synthesis protocol using NaBH_4_.

The effects of the concentrations of HCl, AgNO_3_, AA, and NaBH_4_ in the growth solution are interrelated, making it difficult to categorically identify changes as the result of a single variable. However, the impact of the introduction of oleate on the other components was not well understood; hence, we performed studies on each of these in the presence of oleate. Each component is discussed in the order in which they are added to the growth solution.

### 3.1 Effect of Silver Nitrate Concentration

The presence of silver ions is essential to the formation of single-crystalline AuNRs. Silver plays two key roles in the formation process, it is critical to the initial symmetry breaking in the formation of nascent nanorods, and beyond this it is crucial to regulating the growth on the {110} facets along the sides of AuNR through simultaneous reduction of Ag onto the surface by AA and it’s removal through galvanic replacement by Au leading to continued anisotropic growth.^29,30^ Variation of [Ag] in the growth solution should thus have a strong effect on the aspect ratio of synthesized nanorods.

**Figure 1:**
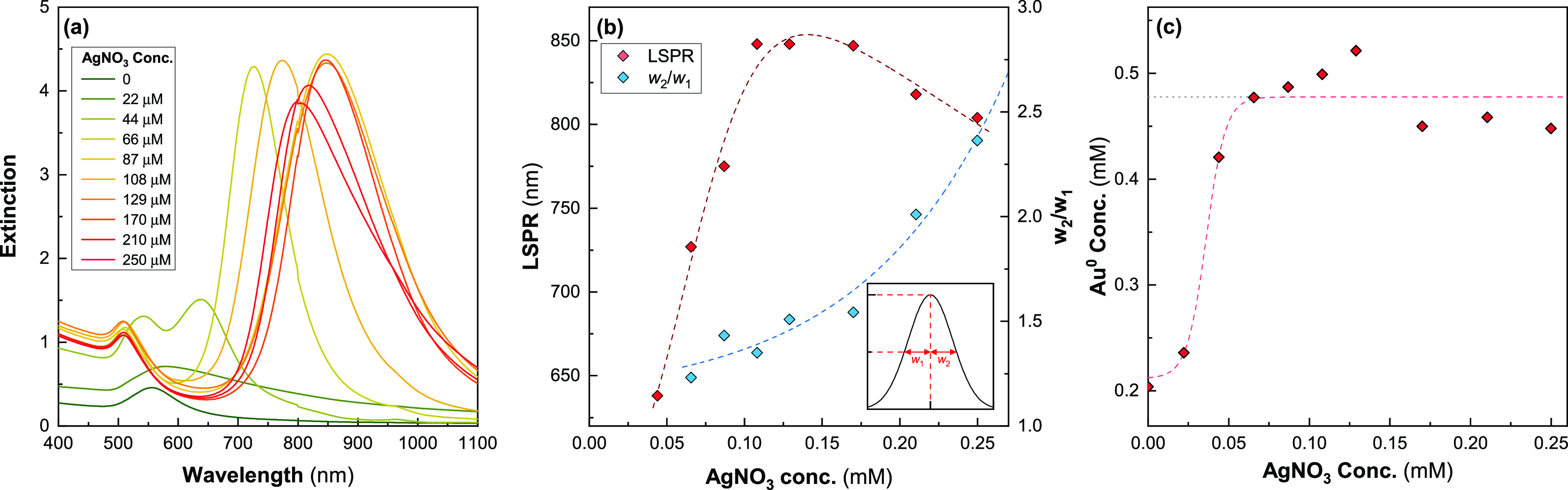
Effect of varying [Ag] on AuNR spectra ([CTAB] = 48 mM, [oleate] = 15 mM) after 4 h. (a) UV–vis spectra at different values of [Ag]. (b) λ_LSPR_ as a function of [Ag]. Also plotted is *w*_2_/*w*_1_, which is the ratio of the widths at half maximum above and below λ_LSPR_ (see inset). (c) [Au^0^] after reaction completion calculated from *A*_400nm_. Results are fitted with a sigmoid.

We investigated the impact of the presence of Ag ions by varying [Ag] between 0 and 250 μM in a growth solution containing [CTAB] = 48 mM and [oleate] = 15 mM. UV–vis spectra of AuNRs synthesized under these conditions are shown in Figure 1a. In the absence of AgNO_3_, we observe only a single peak with a maximum at ∼560 nm, indicating that only gold nanospheres are present as seen in other reports in the literature.^29^ At [Ag] = 22 μM, we observe a single peak with a maximum at ∼580 nm with a tail extending out into the near-infrared, suggesting a population dominated by nanospheres but containing some anisotropic gold nanoparticles. There then appears to be a threshold, between 22 and 44 μM (Au/Ag = 22.7 and 11.4, respectively), above which a second well-defined peak in the near-infrared becomes apparent, consistent with the longitudinal surface plasmonic resonance (LSPR). Combined with the lack of any peak at ∼560 nm associated with non-rod-like side products, it seems that above this threshold the Au is almost completely in the form of AuNRs. Figure 1b shows that the peak wavelength of the LSPR (λ_LSPR_) increases with [Ag] until it reaches a maximum value of ∼890 nm at [Ag] = 108 μM (Au/Ag = 4.6). This shows that raising [Ag] increases the aspect ratio of the AuNRs. This trend has been widely observed elsewhere in the literature (e.g., refs (13), (14), (18), (30), (38–42)). Once [Ag] is increased above ∼108 μM, λ_LSPR_ decreases and the LSPR peak becomes increasingly asymmetric as indicated by the increase in the ratio of the upper half-width maximum to the lower half-width maximum (*w*_2_/*w*_1_). This ratio is an indirect measure of the polydispersity resulting from increasingly large populations of higher aspect ratio AuNRs (Figure 1b). It was not possible to fit peaks where the transverse surface plasmon resonance (TSPR) and LSPR peaks overlapped significantly; hence, only the data for [Ag] ≥ 66 μM (Au/Ag = 7.6) is presented. The LSPR peak becomes increasingly shifted toward longer wavelengths. This is observable in the spectra of other publications, although normally it is not commented upon (i.e., ref (43)). It presumably results from the {110} stabilizing role played by AgNO_3_, which makes the formation of higher aspect ratio AuNRs more preferable; however, the limitations of this growth system seem to prevent these from forming with high uniformity.

The spectra of samples containing [Ag] < 66 μM show an incomplete reduction of the gold salt after 4 h, and these spectra continued to evolve over the following 24 h (Figure S2). Thus, the growth kinetics of the AuNRs were slowed at lower [Ag]. There were no further changes in these spectra after an additional 24 h. It also seems that Ag is acting as a limiting reagent in this system. The final *A*_400nm_ values indicate that there is a noticeable reduction in the fraction of ionic Au that was completely reduced at these lower concentrations (Figure 1c). Ag behaving as a limiting agent is a surprising result, because Ag does not function as a reducing agent in this system and is only expected to affect the geometry of the particles. To our knowledge, both these observations have not been previously reported in the literature. Most reports suggest that the fraction of reduced Au is either independent of [Ag],^39,40^ or increases with decreasing [Ag].^13,38,42^ It is apparent based on these measurements is that [Ag] between ∼44 and ∼170 μM is a reliable parameter to control λ_LSPR_. Above this range, the polydispersity rapidly increases as an increasingly large population of higher aspect AuNRs form.

### 3.2 Effect of Hydrochloric Acid Concentration

HCl is primarily included in the growth mixture to control the reaction pH. The redox potential of AA is pH sensitive, and must be used at a pH where it can only reduce Au^+^ in the presence of an Au^0^ surface to ensure AuNR growth without secondary nucleation. Additionally, it also slows the reaction kinetics allowing more homogeneous formation of AuNRs. For CTAB-only growth protocols, typically a value of [HCl] around 14 mM (pH ∼1.5) is required to prevent Au^0^ nucleation upon AA addition.^14^ In our system the inclusion of the second surfactant, sodium oleate, makes the growth mixture more basic (solutions of sodium oleate above its critical micelle concentration typically have a pH of ∼9.8).^44^ Thus, the inclusion of oleate decreases the reduction potential and increases the reaction kinetics.^45−47^ [HCl] must be increased accordingly to prevent nucleation by AA and to control the reaction kinetics. In the case of an oleate-CTAB mixture, we reported previously that [HCl] ∼ 60 mM was required to produce the same result.^23^

**Figure 2:**
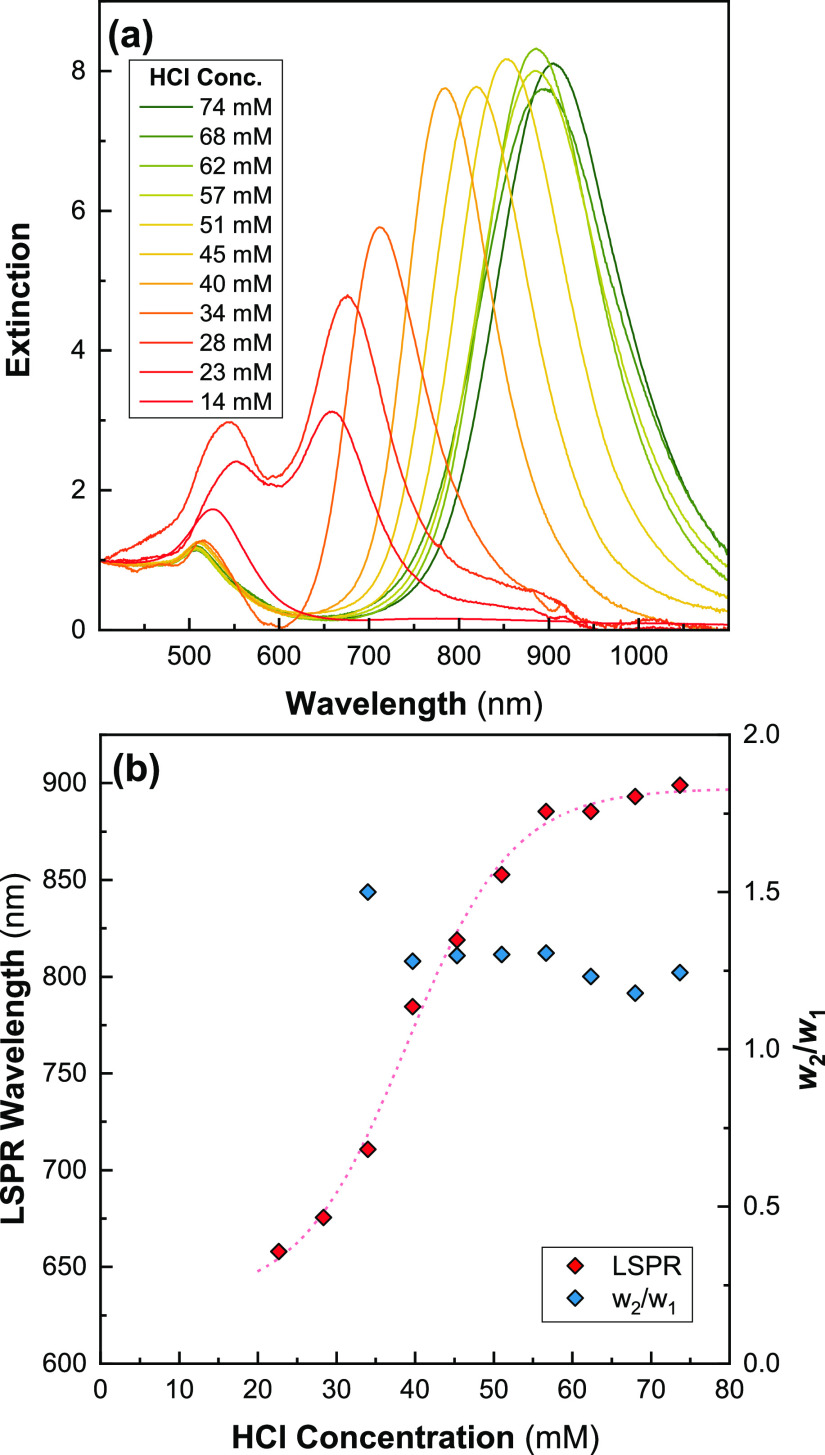
Effect of varying [HCl] on AuNR spectra ([CTAB] = 48 mM, [oleate] = 12.5 mM) (a) UV–vis spectra normalized to *A*_400nm_. Low [HCl] (≲30 mM) leads to high AuNS populations; increasing [HCl] further leads to a red shift in λ_LSPR_ up to around [HCl] ∼ 58.5 mM. (b) λ_LSPR_ as a function of [HCl], demonstrating the increase in λ_LSPR_ with increasing [HCl]. This behavior begins to plateau above [HCl] ∼ 59 mM. Also plotted is *w*_2_/*w*_1_ for spectra with LSPR well separated from the transverse peak. The data point associated with [HCl] = 28 mM has been omitted as no LSPR peak was visible.

Hence, the effect of changing [HCl] was explored by preparing a single growth solution containing [CTAB] = 48 mM, [oleate] = 12.5 mM, and [Au] = 0.5 mM. This was then aliquoted into 11 separate 10 mL batches, in which [HCl] was varied between 23 and 74 mM, followed by the two reducing agents. The spectra of the synthesized AuNRs and the respective LSPR positions are shown in Figure 2.

In the UV–vis spectra, increasing [HCl] above 28 mM leads to high yields of AuNRs evident from relatively low absorbance around 520 nm. Increasing [HCl] leads to a redshift in λ_LSPR_. The changes in the height of the peak are in line with what is expected from Gans’ solution,^55^ due to decreased plasmonic damping by interband transitions above 600 nm, suggesting a high rod yield. The redshift in this peak appears to stop once [HCl] is increased above 54 mM (Figure 2b). HCl thus offers a method to fine tune the λ_LSPR_ of synthesized AuNRs. The asymmetry in the LSPR peak measured by *w*_2_/*w*_1_ remains approximately constant around 1.25 above [HCl] = 34 mM, suggesting that the polydispersity is not affected significantly by [HCl] above this value. For all subsequent syntheses, [HCl] = 57 mM was used. It is worth noting that variations in pH will still occur for other surfactant compositions due to the limited buffering capacity of the growth solution.

### 3.3 Effect of Ascorbic Acid Concentration

Ascorbic acid (AA) is the primary reducting agent during the synthesis and changes in its concentration directly affect the kinetics of AuNR formation. In the binary surfactant system used here, the presence of oleate complicates matters. Oleate is a mild reducing agent, present at a higher concentration than AA and at 30 °C can reduce Au^3+^ to Au^+^. At higher temperatures (≳50 °C), the oleate—CTAB mixture alone can nucleate particles (i.e., without HCl, AA, or NaBH_4_).

**Figure 3:**
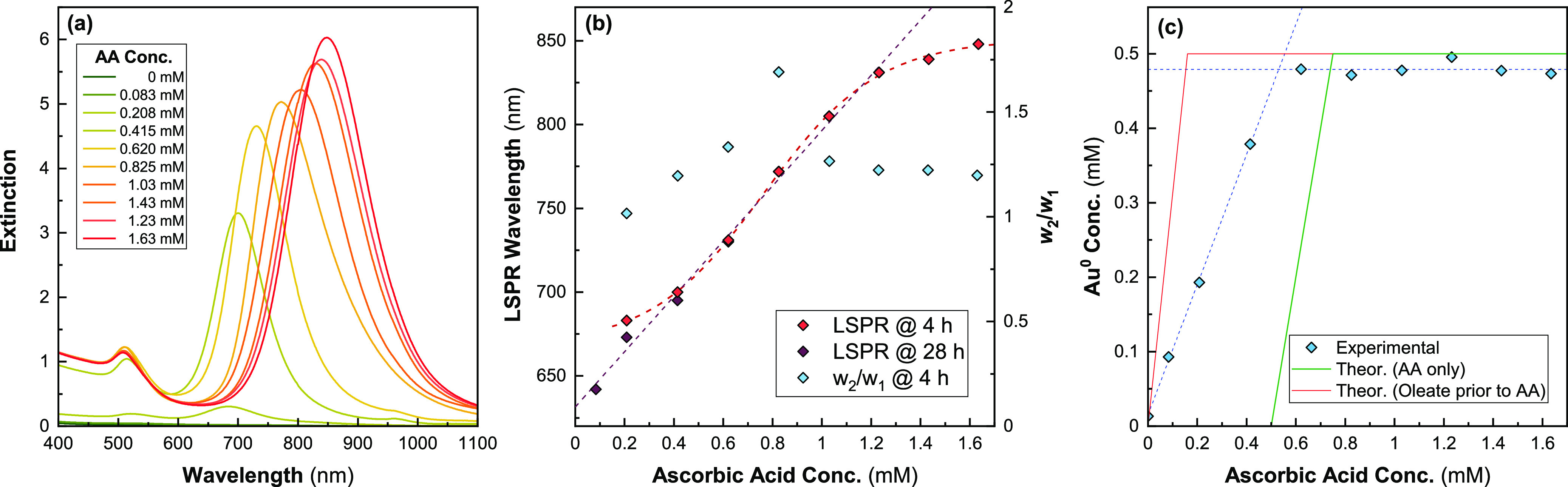
Effect of varying [AA] on AuNR spectra ([CTAB] = 48 mM; [oleate] = 15 mM). (a) UV–vis spectra of AuNRs synthesized using different values of [AA] 4 h after the addition of NaBH_4_. LSPR peaks were not visible for the 0 and 0.083 mM samples. The 0.21 and 0.42 mM samples did not completely reduce all Au^1+^ in solution based on *A*_400nm_ (b) λ_LSPR_ as a function of [AA]. The values at 4 and 28 h after NaBH_4_ addition are presented as separate series. A general trend of increasingly redshifted λ_LSPR_ can be seen with increasing [AA]. The values at 4 h are fitted with a sigmoid and the values at 28 h [AA] < 1.43 mM show a linear trend. Also plotted is *w*_2_/*w*_1_ for spectra with a LSPR peak, which is well separated from the transverse peak. (c) [Au^0^] after 28 h calculated from *A*_400nm_. The theoretical yield assuming perfect reduction of Au^3+^ only by AA is also plotted in green and assuming a complete reduction of Au^3+^ to Au^+^ by oleate before AA addition.

To investigate the effects of AA in this synthesis, a single growth solution, containing [CTAB] = 48 mM and [oleate] = 15 mM, was split into several 10 mL batches following the same protocol as above and [AA] was varied between 0 and 1.63 mM. The resulting spectra and change in the LSPR are given in Figure 3a,b, respectively. λ_LSPR_ increased linearly with increasing [AA] up to ∼1.43 mM, where this trend began to plateau. The synthesized rods had narrow symmetric peaks, as demonstrated by the relatively consistent *w*_2_/*w*_1_ values around ∼1.25 (Figure 3b). All spectra had high *A*_LSPR_/*A*_TSPR_ ratios, with no evidence of non-rod-like nanoparticles. The reaction did not progress at all in the absence of AA, consistent with oleate being incapable of reducing the Au^+^ onto the particles under these reaction conditions.^21^ Values of [AA] < 0.62 mM incompletely reduce the Au salt, 4 h after the addition of NaBH_4_. Further incubating at 30 °C for an additional 24 h (*t*_total_ = 28 h) demonstrated that this resulted from AA acting as a limiting reagent below 0.62 mM. However, the spectra of samples with lower concentrations continued to evolve after 4 h, due to the slower reaction kinetics associated with lower [AA] (Figure S3). This is most clear in the case of [AA] = 83 μM, where two previously undetectable peaks emerged in the spectrum after 28 h. For [AA] = 208 μM, there was a large increase in extinction at all wavelengths in the same period, suggesting that most of the Au^+^ in solution was reduced during this time. All reactions appear to have been completed after 28 h and no further changes in the spectrum were observed after this point.

As shown in Figure 3c, the near-complete reduction is observed (>96%) for all samples above [AA] ≳ 0.5 mM. Below this not all the gold salt is reduced to Au^0^, suggesting that AA is the limiting reagent for [AA] ≲ 0.5 mM. The stoichiometric ratio of Au^3+^/AA for complete reduction to Au^0^ is 1.5 without the presence of other reductants (i.e., [AA] = 0.75 mM);^40^ hence, not all the reduction in this system is being caused by AA. This can be seen from the way our data deviate from the theoretically expected curves for AA acting as the sole reducing agent in Figure 3c. Oleate thus appears to facilitate some reduction of the Au^3+^ to Au^+^. This reduction is partially provided by the AA, as the observed yields also do not match that expected for a synthesis where all the reduction of Au^3+^ to Au^+^ is performed by oleate either. At the intersection of the two linear fits ([AA] = 0.53 mM, i.e., where complete reduction to Au^0^ should occur) would require ∼44% reduction of Au^3+^ to Au^+^ by oleate. The percentage of reduction done by oleate will increase with decreasing [AA]. This fraction is dependent on the time elapsed between the mixing of oleate and Au^3+^, and the later addition of AA, similar results have been reported for reduction with salicylic acid.^15^ Allowing more time to elapse should result in further reduction by oleate because it exists in a 30× molar excess relative to Au. This may be desirable as it would increase the reproducibility of this synthesis by minimizing variation in the concentration of unreacted AA at nucleation resulting for differences in the time between the addition of reactants. These results are also consistent with the comproportionation scheme for AA suggested by Scarabelli et al.^15^

The observed changes in λ_LSPR_ with [AA] seen here support reports elsewhere that the aspect ratio increases with increasing [AA] but begins to drop at higher values of [AA].^48,49^ However, contradictory reports exist in the literature suggesting that increasing [AA] should cause a decrease in the aspect ratio (i.e., refs (50) and (51)). Increasing [AA] will substantially speed up the reaction and appears to have little impact on the quality of the product based on their spectra alone. This decreased synthesis time could potentially enable this method to be used in continuous flow methods, enabling syntheses to be substantially scaled up for industrial production.

### 3.4 Effect of Sodium Borohydride Concentration

NaBH_4_ is a strong reductant used to induce nucleation in this protocol and plays the same role as the seed solutions added during seeded monocrystalline AuNR syntheses.

**Figure 4:**
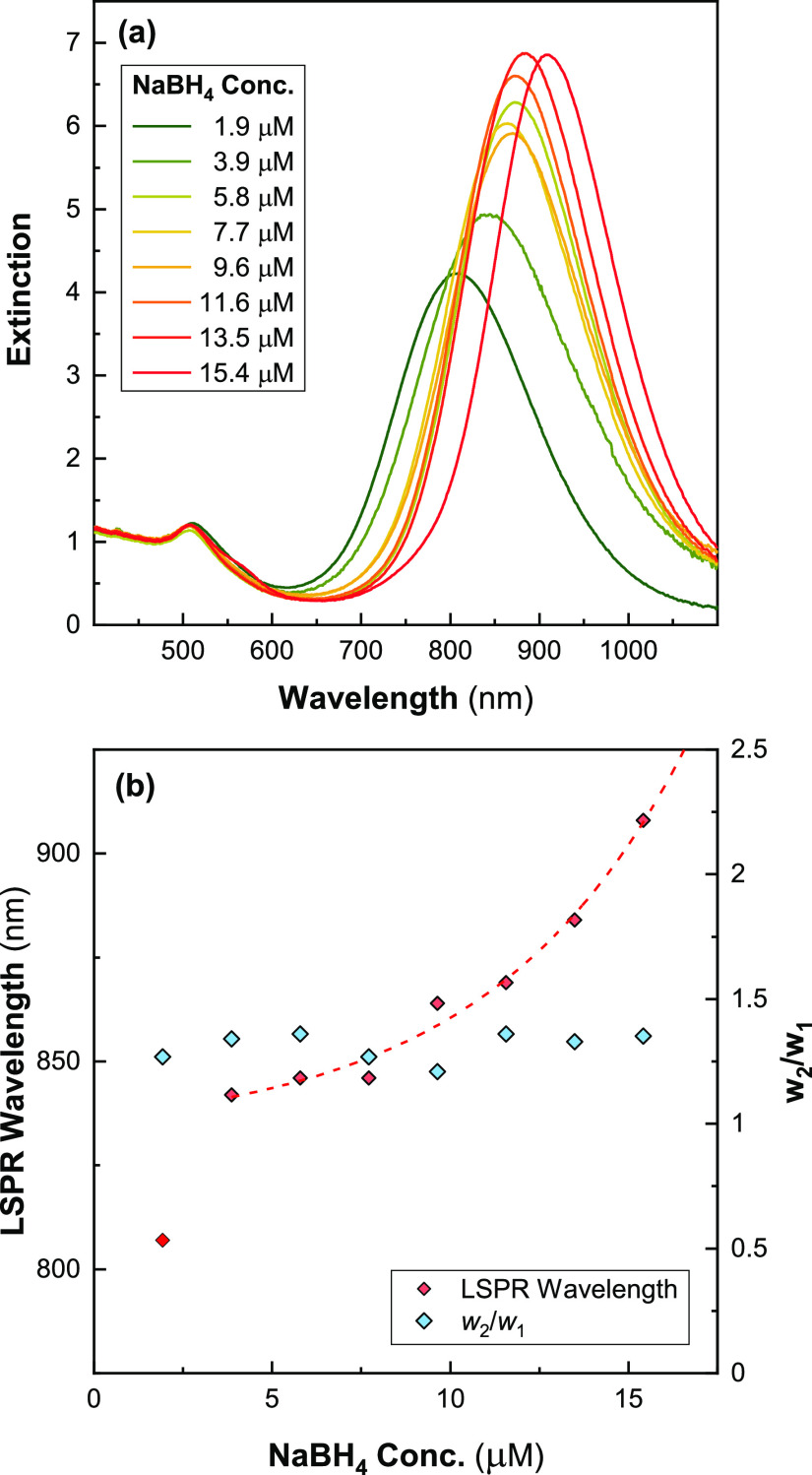
Effect of varying [NaBH_4_] on AuNR spectra ([CTAB] = 48 mM; [oleate] = 12.5 mM). (a) UV–vis spectra showing a general trend of increasingly redshifted λ_LSPR_ values can be seen with increasing [NaBH_4_]. Low [NaBH_4_] can be seen to lead to broader LSPR peaks (more polydisperse). (b) λ_LSPR_ as a function of [NaBH_4_], demonstrating the increase in λ_LSPR_ with increasing [NaBH_4_]. The red data point associated with 1.9 μM is not included in the fit. Also plotted is *w*_2_/*w*_1_ for spectra with a LSPR peak well separated from the transverse peak.

To explore the effect of varying [NaBH_4_] this in our system, a single growth solution was prepared containing [CTAB] = 48 mM, [oleate] = 12.5 mM, and [Au] = 0.5 mM. This was aliquoted into 10 mL batches and [NaBH_4_] varied between 1.9 and 15.4 μM. A clear increase in λ_LSPR_ was seen with increasing [NaBH_4_], indicating that higher aspect ratio AuNRs were synthesized (Figure 4a,b). λ_LSPR_ for [NaBH_4_] = 1.9 μM did not match the trend seen for higher [NaBH_4_]. It is unclear whether this resulted from some new particle growth regime at low [NaBH_4_]. Given that there is expected to be an increased number of AuNRs with increasing [NaBH_4_], and the same finite reservoir of Au ions, it is assumed that these higher aspect ratio AuNRs must be of reduced diameter.

All LSPR peaks had relatively low asymmetry as measured by *w*_2_/*w*_1_, remaining consistently around a value of ∼1.3, suggesting that the polydispersity was low and [NaBH_4_] had little impact on the AuNR polydispersity. For all spectra, a near complete reduction of the Au^3+^ occurred, based on the final *A*_400nm_ being close to 1.2 ([Au^0^] ∼ 0.5 mM) for all spectra.

There are conflicting reports of the effect of changing [NaBH_4_] (or equivalently concentration of seeds), with multiple reports that an increase leads to a blueshift in λ_LSPR_^17,37,52,53^ and several others observe a redshift.^14,18,19,39,42,54^ Our results match the observations of the latter group reporting a redshift. There have been some suggestions that the effect of [NaBH_4_] is highly dependent on the pH and relative concentrations of other components in the growth solution.^14,17^ Consistent with our observations, other protocols using oleate have observed a redshift; however, this trend seems to be reversed at higher pH.^20,21^ It is not clear which processes are driving these changes in the AuNR aspect ratio. Increasing [NaBH_4_] reduces the size of each particle, as there is less Au^3+^ per nucleation. However, this does not translate into a simple relationship with the particle aspect ratio.

Our results demonstrate that [NaBH_4_] can be used to control the aspect ratio of the synthesized AuNRs, it seems that 7.25 μM is a sensible value of [NaBH_4_] which minimizes variability between batches, because it falls in a section of the curve that is less sensitive to changes in [NaBH_4_]. The respective gradients at low (5.8 μM) and high (15.4 μM) [NaBH_4_] are ∼1.9 and ∼9.5 nm μM^–1^.

### 3.5 Kinetic UV–Vis Spectroscopy

In our previous work, we showed that increasing [oleate] in this system causes a blueshift in λ_LSPR_ with an accompanying increase in the length and diameter.^23^ To further understand the impact of [oleate] on the evolution of the AuNRs, we monitored their spectra during the synthesis. Spectra were taken at 2 min intervals and the change in λ_LSPR_, maximum extinction, and the full width at half-maximum (FWHM) of the LSPR peak were recorded, as well as *A*_400nm_. From this, we can assess the concentration of reduced Au^0^ in solution and approximate the average aspect ratio and monodispersity of the synthesized AuNRs.

**Figure 5:**
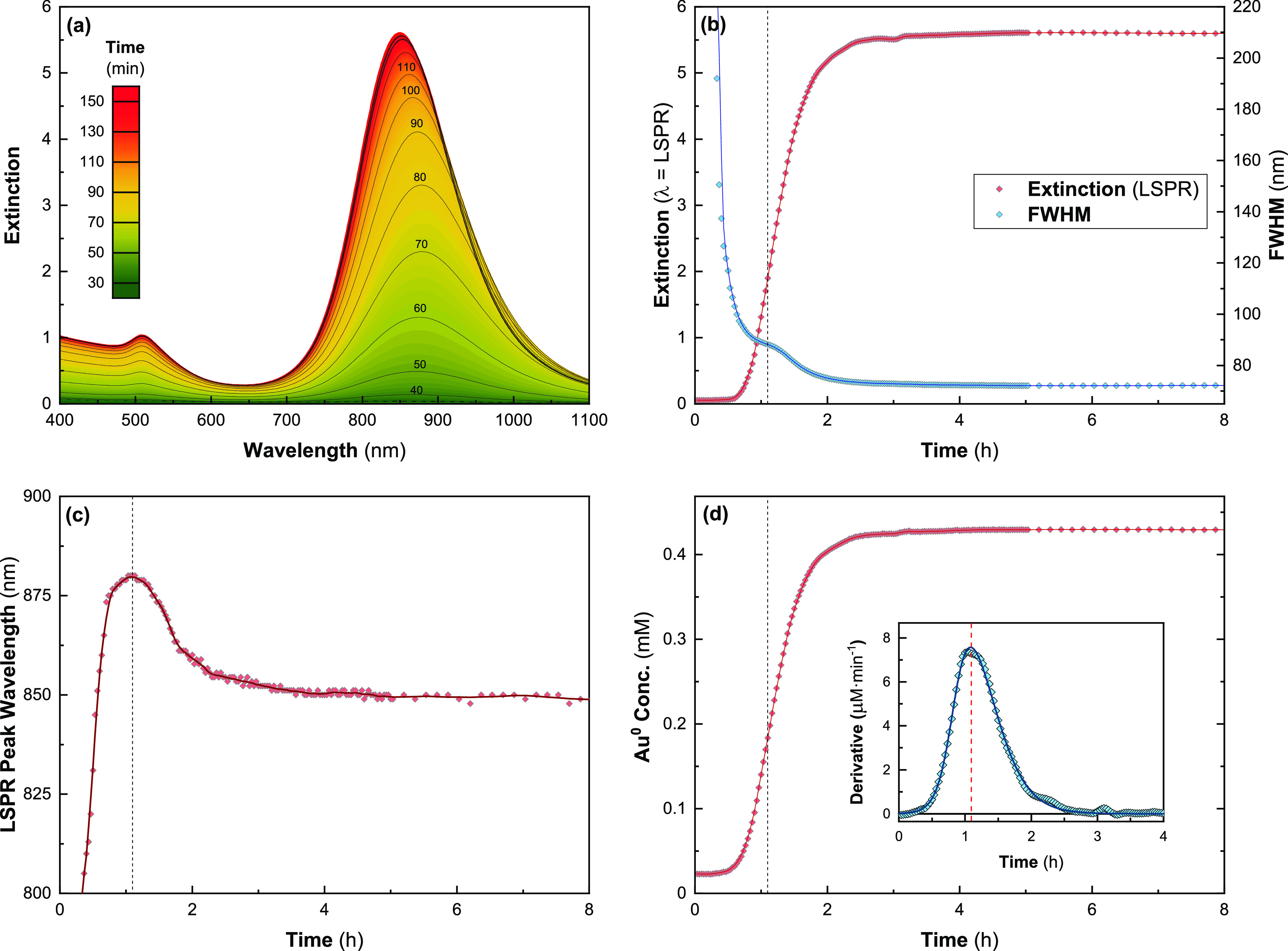
Kinetic UV–vis monitoring of AuNR synthesis ([CTAB] = 48 mM; [oleate] = 12.5 mM). (a) Kinetic UV–vis spectra of AuNR synthesis taken at 2 min intervals. (b) Measured extinction at λ_LSPR_ and FWHM of the LSPR peak as determined by a Gaussian fit. (c) λ_LSPR_ as a function of time; the maximum of this curve at ∼65 min has been marked by a dashed line in (b,d). (d) [Au^0^] calculated from *A*_400nm_. Inset shows the derivative given in μmol per L of Au^+^ reduced per min.

Spectra were taken at a range of [oleate] with [CTAB] fixed at 48 mM. The blueshifting of the LSPR peak with increasing [oleate] was previously observed (Figures 5 and S4–S7) with the same corresponding decrease in aspect ratio as observed by TEM (Figures S8–S12). Figure 5a shows the evolution of a spectrum of these samples using a growth solution with [CTAB] = 48 mM and [oleate] = 12.5 mM over 8 h. The reaction completed within ∼4 h and the LSPR peak evolved throughout this period. Initially becoming apparent from the background after ∼20 min and rapidly redshifted until around 1 h, after which it slowly blueshifted until all Au^3+^ was exhausted (Figure 5c). Other researchers have reported this trend in λ_LSPR_ elsewhere.^15,29,33,54^ It suggests an initial anisotropic growth phase during which the AuNR aspect ratio rapidly increases, followed by a second, more isotropic growth phase in which the aspect ratio slowly reduces.^17,19,29^

During our experiments, there was an observable increase in *A*_400nm_ during both phases, indicating that Au^+^ was still being reduced throughout (Figure 5d). This contrasts with observations by Edgar et al., who using a CTAB-only synthesis (no oleate) found that the reduction in λ_LSPR_ occurred after Au^+^ reduction has ceased. They thus concluded that this blueshift could not be caused by anisotropic growth and instead must be caused by the reshaping of the AuNRs themselves, primarily through the modification of the tips from sharp crystalline facets to more rounded ends.^33^ However, this cannot be the case during our synthesis as Au reduction continues throughout the observed blueshift in λ_LSPR_. In our experiments, the longest λ_LSPR_ was typically achieved at, or just before, the point of fastest Au^+^ reduction, and Au^0^ continued to be deposited onto the particles for a significant period thereafter (Figures 5 and S4–S7). It is therefore likely that Au^0^ continued to be deposited on the AuNRs in a manner which resulted in a change in the aspect ratio, driving the observed shift in λ_LSPR_. The studies mentioned above only use CTAB in their growth solution, completing within 30–40 min,^33,54^ by comparison the reactions here take at least 2 h to complete, often taking significantly longer than this. Processes that drive end-cap reshaping, such as oxidative etching or adatom migration, are unlikely to be able to compete with the rate of Au reduction onto the AuNRs seen here. There is generally little change in λ_LSPR_ once *A*_400nm_ saturates in most cases (except for [oleate] = 7.5 mM, Figure S4), implying that at high [oleate], tip morphology is largely static after the completion of Au^+^ reduction.

**Figure 6:**
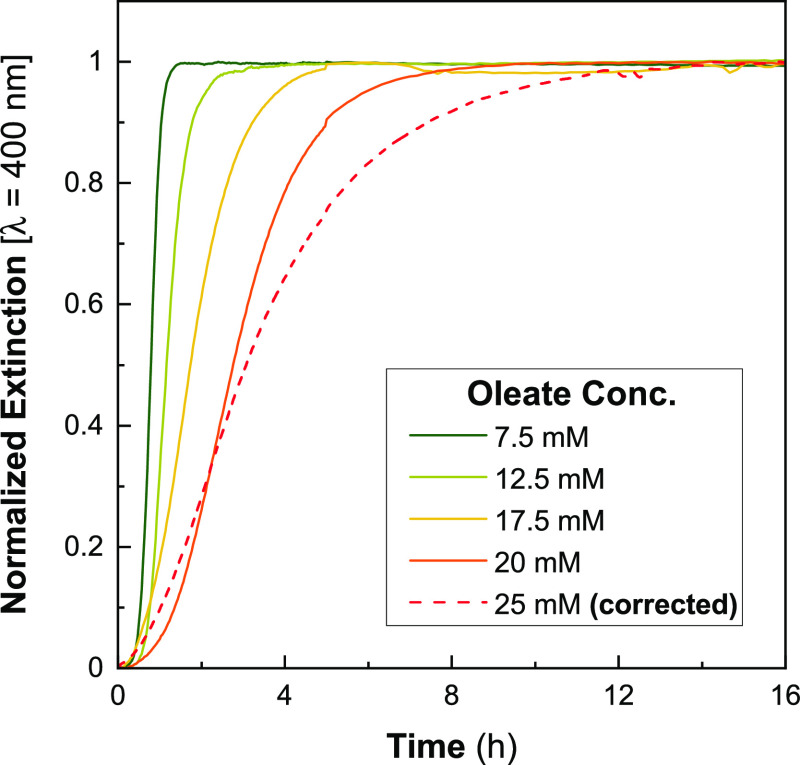
Effect of varying [oleate] on the reaction kinetics of AuNR synthesis. Curves are normalized to the final stable value of *A*_400nm_. Note: the [oleate] = 25 mM curve is not raw data and has been corrected to account for turbidity in the growth solution, see Section S2.2 for details.

The FWHM of the LSPR peak decreased throughout the reaction, suggesting that the polydispersity of the AuNRs consistently dropped throughout the synthesis (Figure 5b). There is a noticeable point of inflection in a number of these curves that occurs ∼90 min after NaBH_4_ addition. It is not clear what causes this, but it seems to be largely independent of [oleate]. Comparing *A*_400nm_ curves between the different values of [oleate] shows a clear trend of the decreasing Au^0^ evolution rate with increasing [oleate] (Figure 6). A possible explanation for this is the increased packing density of the surfactants on the surface of the AuNR. The incorporation of more negatively charged oleate into the positively charged CTAB bilayer increases electrostatic screening between the quaternary ammonium headgroups, increasing surfactant packing and reducing the accessibility of Au^+^ ions to the surface of the AuNR.

In our previous report, we terminated reactions after 4 h; however, the slower reaction kinetics seen here suggest that at high [oleate], the reaction had not completed at this time point.^23^ For instance, in the experiments reported here using [oleate] = 20 mM (Figure S9) resulted in *A*_400nm_ not increasing until ∼6 h after NaBH_4_ addition. This would have meant that ∼25% of the gold precursor was wasted by separating the particles from the growth solution by centrifugation at this point. It also implies that at higher [oleate] we erroneously concluded that the syntheses were failing, when in fact a longer reaction time would have yielded useful AuNRs.

## 4 Conclusions

In the CTAB-oleate AuNR synthesis, we observed dramatic changes in the optical properties of synthesized AuNRs resulting from the variation of numerous components in the growth solution. Increasing [HCl] and [Ag] led to redshifts of λ_LSPR_. In the case of Ag, the optimum concentrations for growth are between ∼80 and ∼180 μM. Above 180 μM, particle distributions become increasingly polydisperse, with asymmetric peaks with strong tails into the near-infrared. Below 80 μM, the yield of Au^0^ was reduced and the reaction kinetics slowed considerably.

Increasing the volume of NaBH_4_ added to the growth solution increased the aspect ratio of synthesized particles. Similarly, increasing the concentration of AA led to an increase in the aspect ratio. It also showed that oleate does a large proportion of the reduction of Au^3+^ to Au^0^ in this synthesis. The timescale for this to complete is longer than the time between the mixing of oleate and Au and the subsequent addition of AA. Hence, in our previous report, both oleate and AA reduced the gold salt. For improved reproducibility, it seems that a good approach would be to allow the oleate to reduce all Au^3+^ to Au^+^, and [AA] adjusted accordingly.

Kinetic spectra of the binary surfactant syntheses showed that they evolve similarly to other single crystalline syntheses. The inclusion of oleate led to changes in the kinetics of the reaction. Higher [oleate] reduced the growth kinetics of the reaction. This meant that some reactions completed over 12 h, rather than the 2 h typically required for other syntheses. Other observations of these experiments suggest that the changes in the spectra are driven almost entirely through the direct reduction of Au onto the particles rather than via other processes causing tip reshaping. Tip reshaping is unlikely here due to there being little change in the optical spectra after gold reduction has ceased.

These observations could help with the design of future syntheses and enable the production of AuNRs with desirable optical properties. We hope that they will further assist in understanding the processes that combine to drive the formation of AuNRs during synthesis.

## Supporting Information Available

Calibration of [Au^0^] from UV–vis extinction; effects of [Ag] on reaction kinetics as measured by UV–vis spectroscopy; effects of [AA] on reaction kinetics as measured by UV–vis spectroscopy; kinetic UV–vis spectra at different [oleate] and TEM micrographs of selected AuNR samples (PDF)

## Abbreviations

*A*_400nm_: absorbance at 400 nm
AA: ascorbic acid
*A*_LSPR_: peak absorbance of the LSPR
*A*_TSPR_: peak absorbanceof the TSPR
AuNR: gold nanorod
CTAB: cetyltrimethylammonium bromide
FWHM: full width at half maximum
λ_LSPR_: peak wavelength of the LSPR
LSPR: longitudinal surface plasmon resonance
TEM: transmission electron microscopy
TSPR: transverse surface plasmon resonance
*w*_2_/*w*_1_: ratio of the upper half-width maximum (*w*_2_) to lower half-width maximum (*w*_1_)
