# Supplementary material for: Controlling the Optical Properties of Gold Nanorods in One-Pot Syntheses
Source: J Phys Chem C Nanomater Interfaces. 2022 Feb 3;126(6):3235–43. doi: 10.1021/acs.jpcc.1c10447 (PMC9007536; doi:10.1021/acs.jpcc.1c10447)
Supplement: Supplementary file 1 — jp1c10447_si_001.pdf [file jp1c10447_si_001.pdf]

Supporting Information: Controlling the Optical Properties of Gold Nanorods in One-Pot Syntheses

*Lucien Roach[1]†, P Louise Coletta[2], Kevin Critchley[1], and Stephen D. Evans[1]**

1. School of Physics and Astronomy, University of Leeds, Leeds, LS2 9JT, UK
2. Leeds Institute for Medical Research, University of Leeds, Leeds, LS2 9JT, UK

* Corresponding author: s.d.evans@leeds.ac.uk

S1. Calibration of Au^0 Concentrations from UV-vis

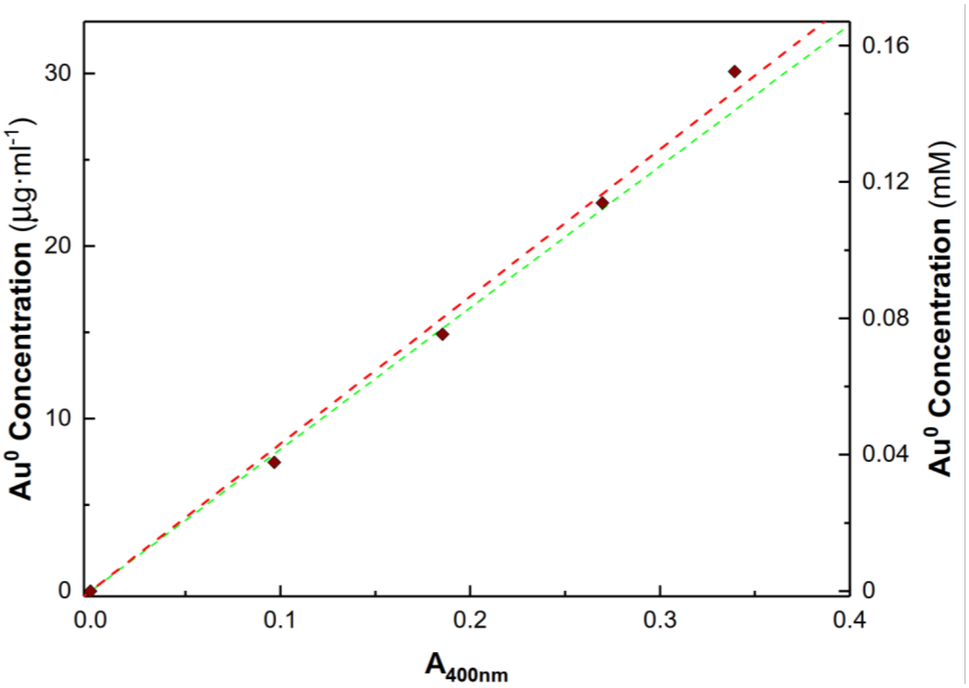

Figure S1. Measured [Au^0] as determined by atomic absorbance spectroscopy as a function of A_{400nm} of the digested solution. The red line is a linear fit, whereas the green line is the published value of ~0.416 mM.

S2. Effects of [*Ag*] on Reactions Kinetics

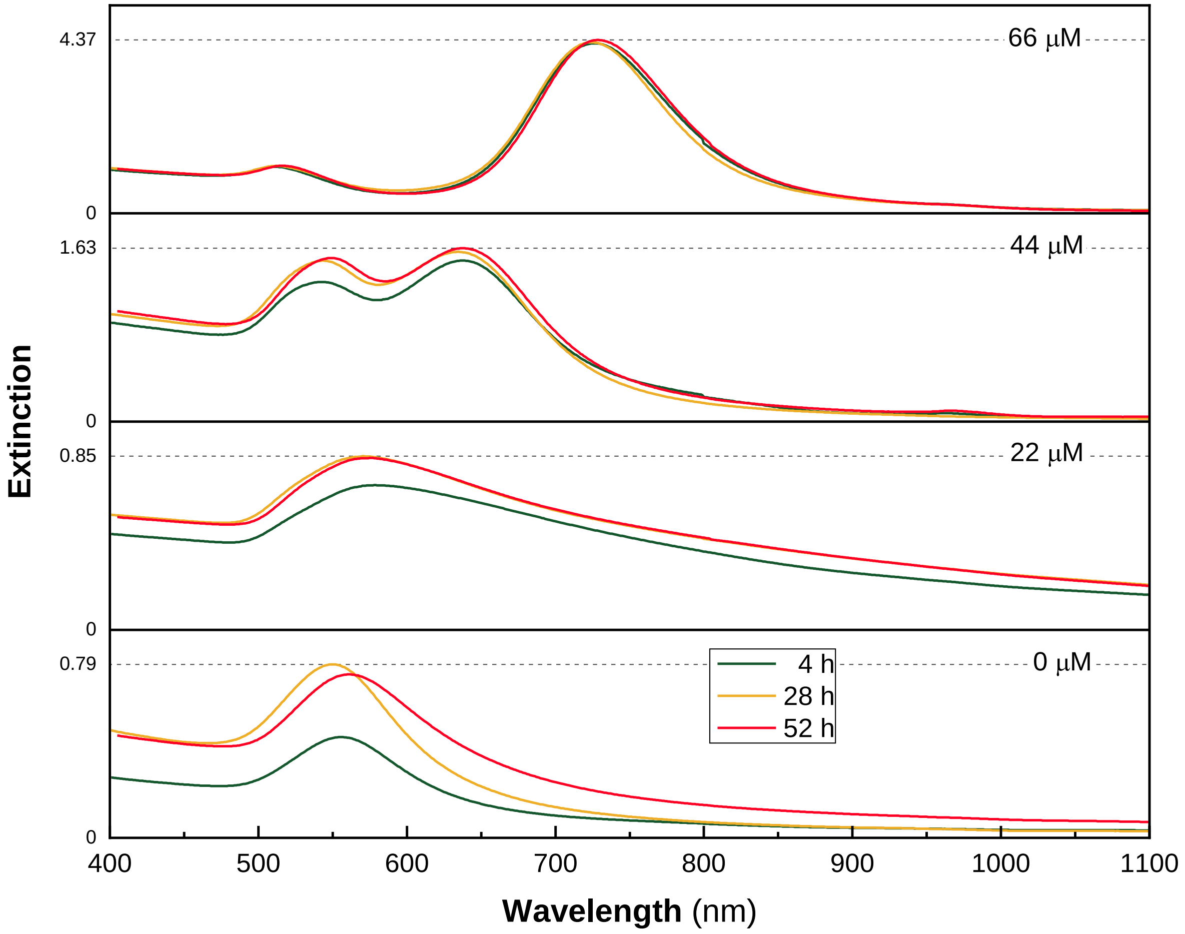

Figure S2. UV-vis extinction spectra of samples at low [*Ag*] as recorded at 4, 28, and 52 h after $NaBH_4$ addition. Samples above [*Ag*] = 66 μM have been omitted as they showed no change after 4 h.

S2. Effects of [*AA*] on Reactions Kinetics

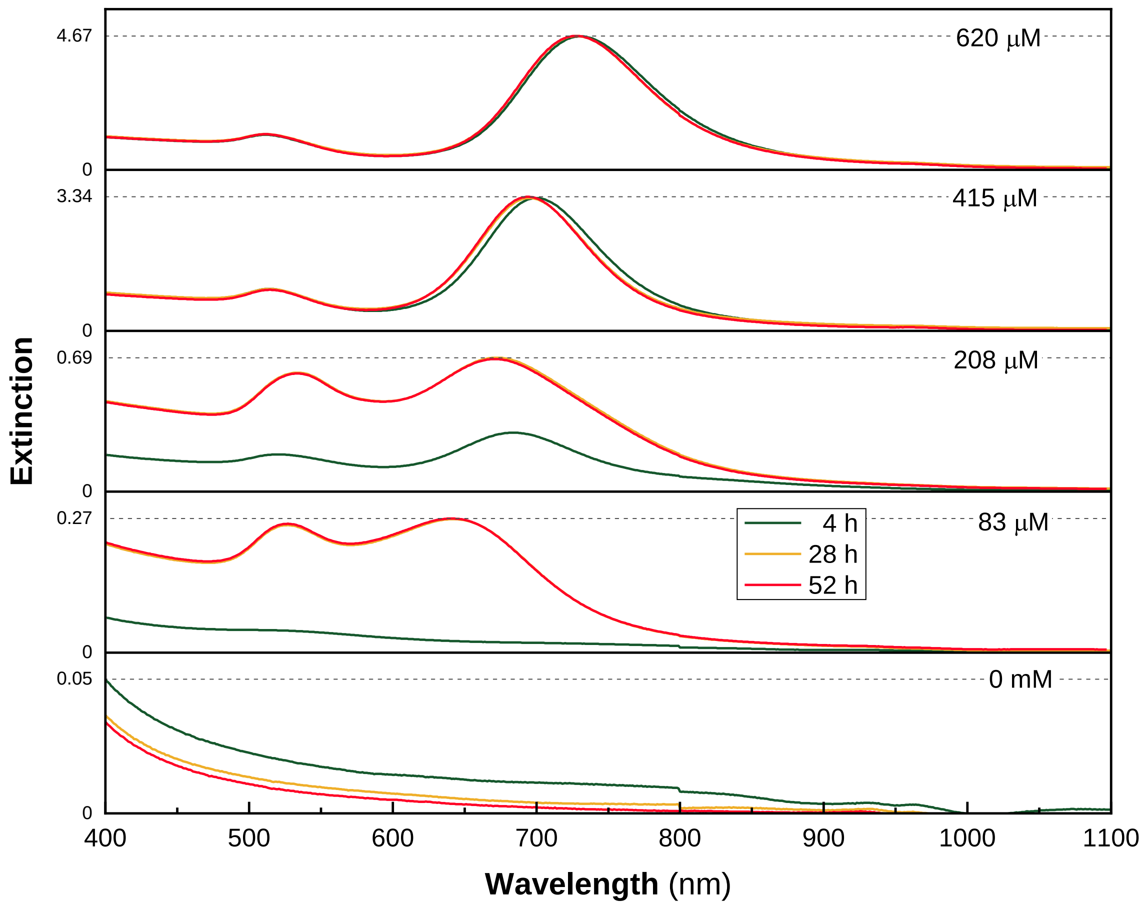

Figure S3. UV-vis extinction spectra. Samples at low [*AA*] recorded at 4, 28, and 52 h after $NaBH_4$ addition. There are substantial changes in the spectra of the 0.083 and 0.21 mM samples after 28 h. There was no formation of AuNRs in the 0 mM sample at any time point. Vertical scale is not consistent between different AuNR batches

S3. Kinetic UV-vis Spectra

Presented here is the analysis of the kinetic spectra discussed in Section 4. For each figure, (a) contains the kinetic UV-vis spectra. (b) contains the extinction at the LSPR peak and FWHM of the LSPR. (c) contains the LSPR peak wavelength as a function of time, and (d) [Au^0] as function of time; inset into this panel is the derivative of the Au^0 concentration giving the rate of Au 0 reduction. The dashed vertical lines in (b)–(d) are the times of peak reduction (blue) and the time of maximum LSPR wavelength (red). In the instances where these two times coincided the line is black

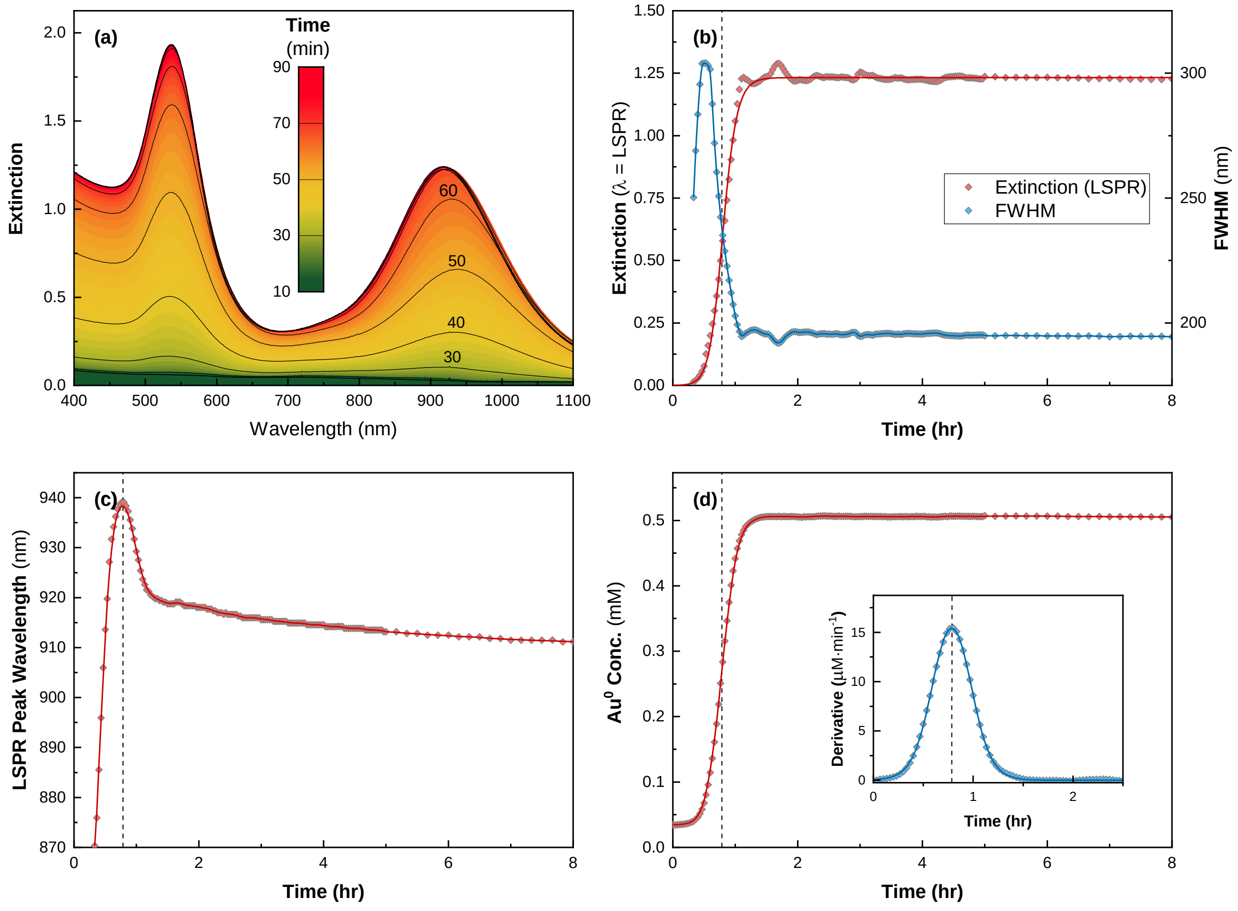

Figure S4. 48 mM CTAB, 7.5 mM oleate

[**48 mM CTAB, 12.5 mM** is given as **Figure 5** in the main paper.]

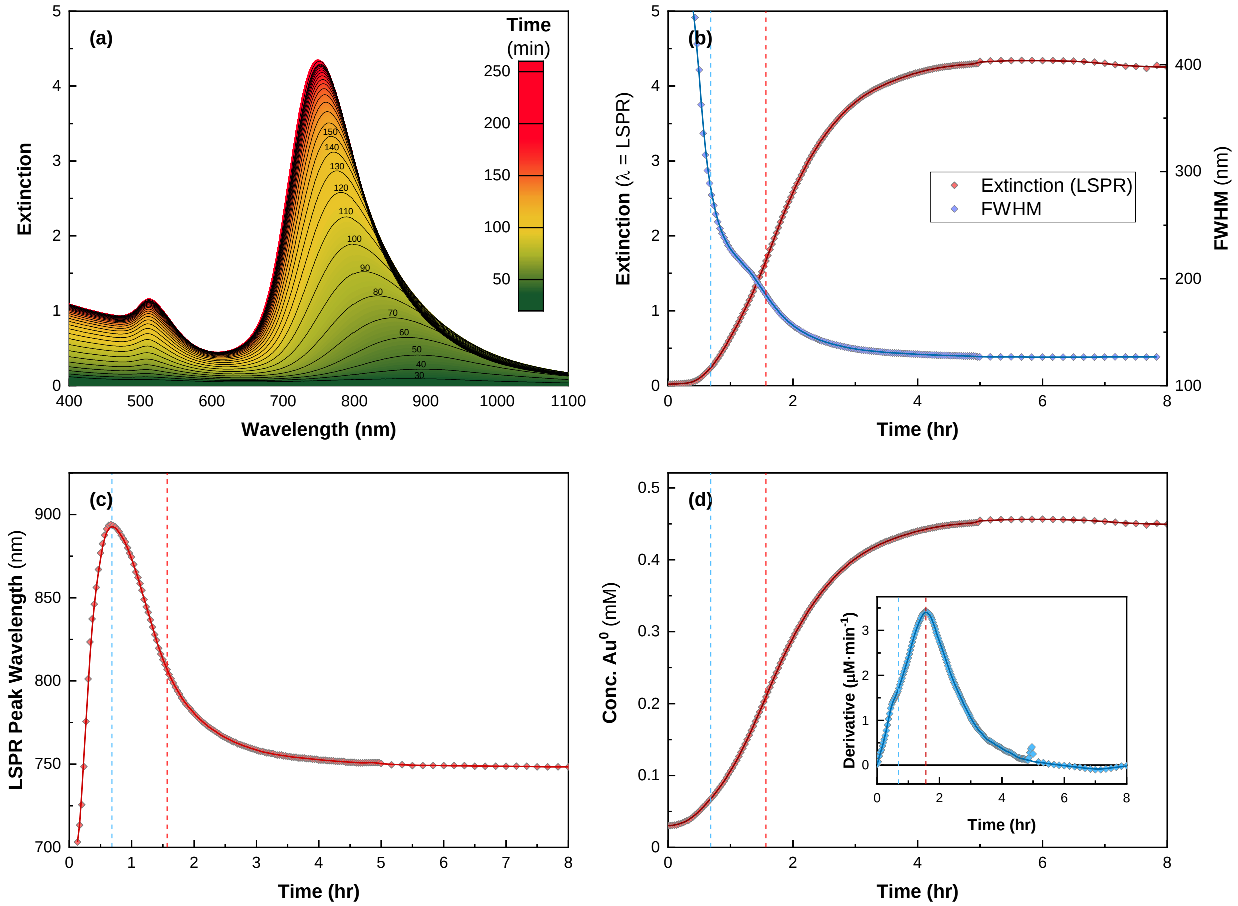

Figure S5. 48 mM CTAB, 17.5 mM oleate

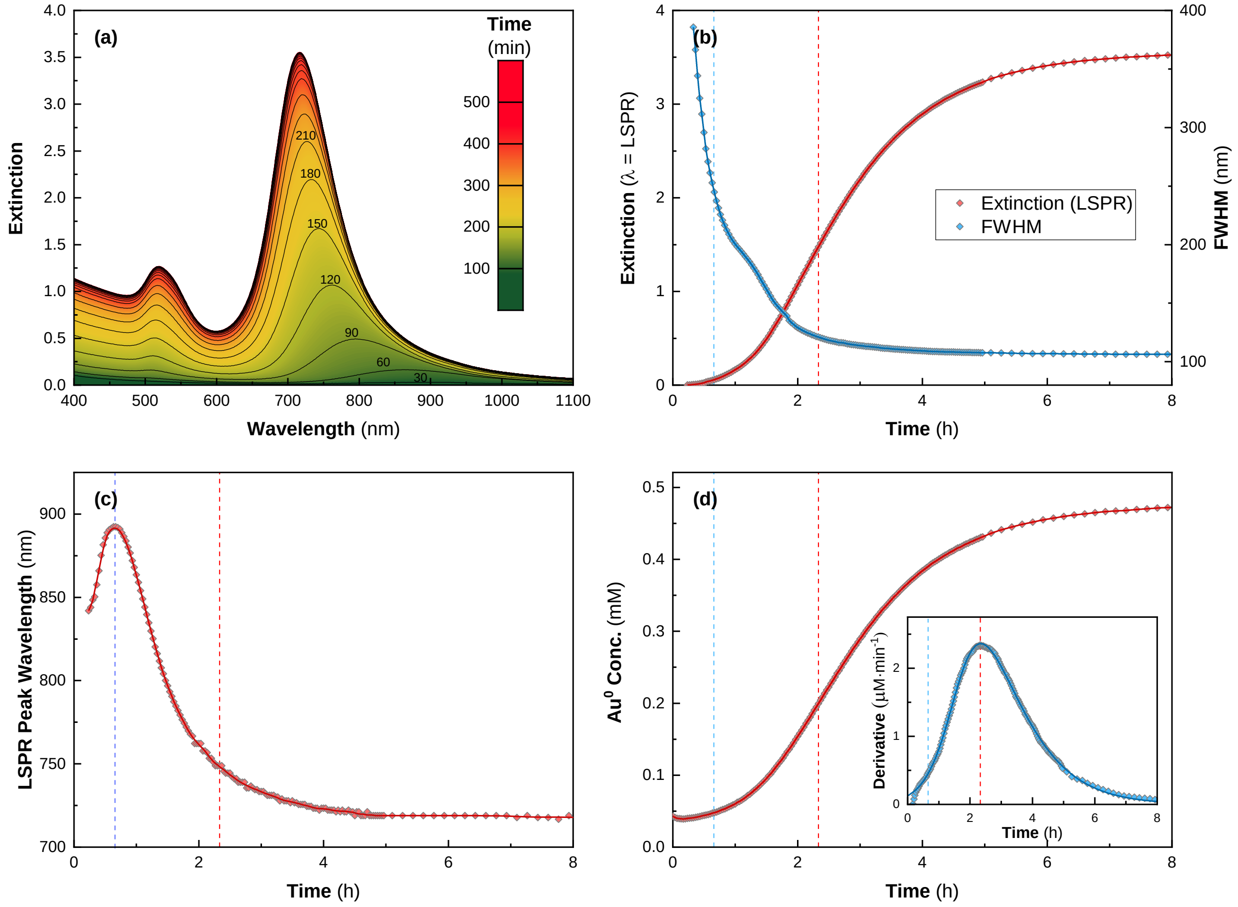

Figure S6. 48 mM CTAB, 20 mM oleate

S3.1 Correction to 25 mM Kinetic UV-vis Spectra

The synthesis solution containing 48 mM CTAB, 25 mM NaOL presented an issue. The growth solution became was cloudy upon preparation indicating the formation of structures that scattered light strongly such as vesicles or bubbles. In the spectra presented in **Figure S4(a)** the formation of plasmonic peaks can be seen, but an additional contribution to the spectrum is apparent, which evolves with time, which severely limits the usefulness of the kinetic spectra. The spectrum of the final product after washing via centrifugation is given as the thick black in this panel, (i.e. surfactant contribution to the extinction has been removed). We cannot reliably extract the LSPR peak extinction and FWHM values from these spectra. In panel **(b)** an attempt at correcting $A_{400\text{nm}}$ has been made. At 1100 nm there is minimal plasmonic contribution and no interband contribution to the extinction hence it is dominated by scattering, the shape of this curve should be similar to the scatter contribution at 400 nm. A correction was made by subtracting $A_{1100\text{nm}}$ from $\text{A}_{400\text{nm}}$ multiplied through by a scaling factor, $c_1 = 2.7$. This scaling factor was arbitrarily selected to give a gradient of zero in the $A'_{400\text{nm}}$ curve after the reaction was finished:

$$A'_{400\text{nm}} = A_{400\text{nm}} - c_1 \cdot A_{1100\text{nm}} \tag{1}$$

This was then multiplied by a second scaling factor $c_2 = A^{(cleaned)}_{400\text{nm}} / A'_{400\text{nm}}(t = 24\text{ h})$:

$$A^{(corr)}_{400\text{nm}} = c_2 \cdot A'_{400\text{nm}} \tag{2}$$

To give a final extinction value of ~1.18 at 400 nm. The recovered values broadly fit in with the trends seen in Figure 6 but **should still be treated with skepticism**. Panel **(c)** presents the position of the peak LSPR wavelength as a function of time (but this has been slightly shifted by the background signal.

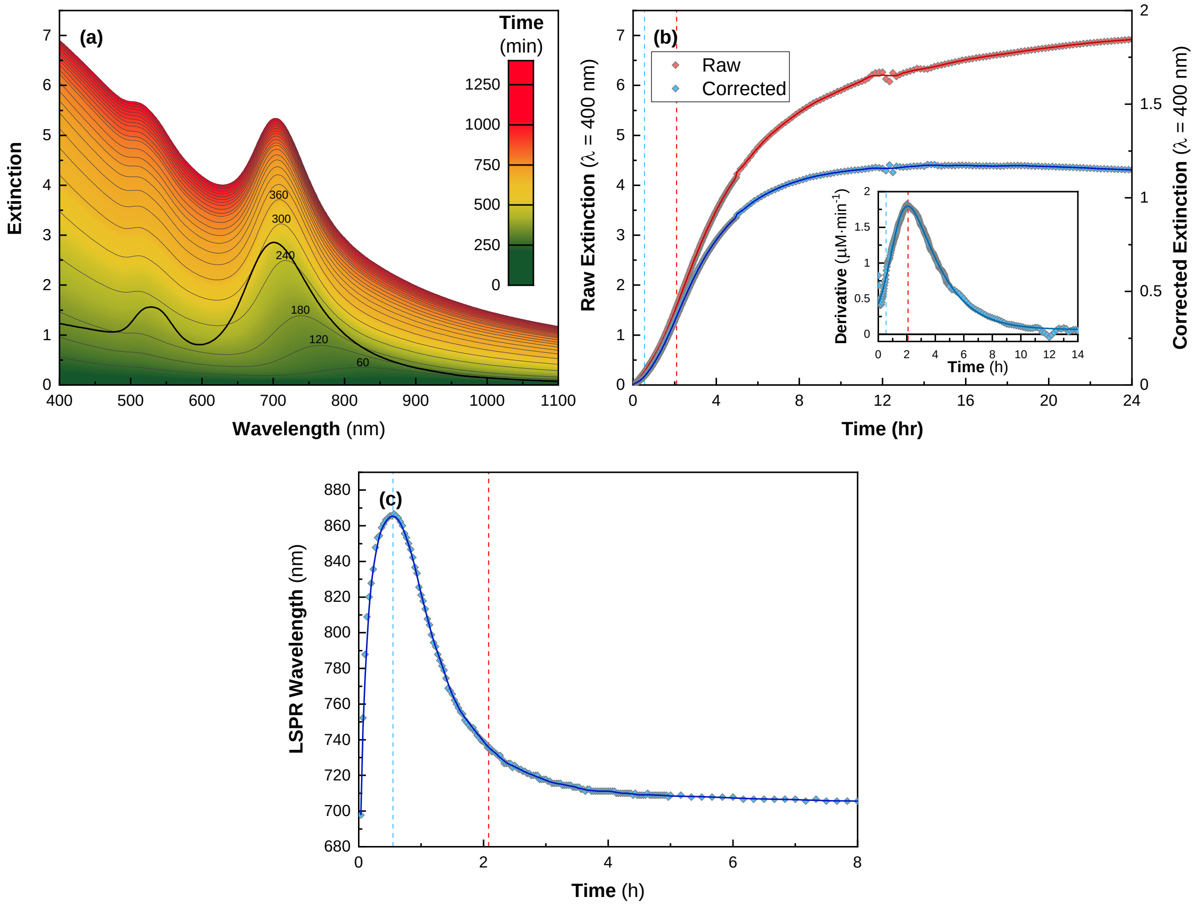

Figure S7. 48 mM CTAB, 25 mM oleate

S3 TEM Images

Additional TEM images from this data set are available through the University of Leeds Data Repository (https://doi.org/10.5518/1082)

S3.1 48 mM CTAB – 12.5 mM NaOL

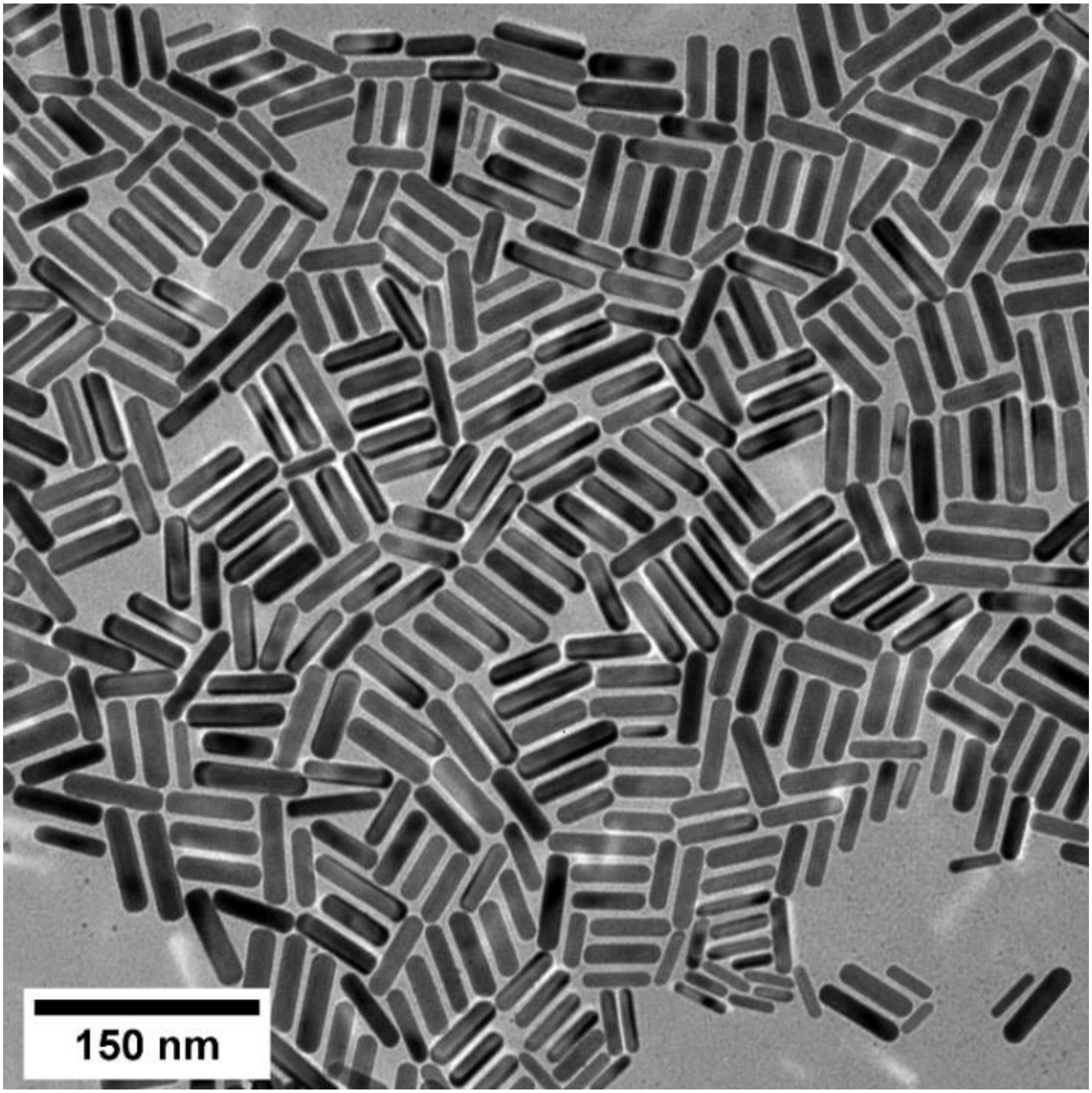

Figure S8. TEM of AuNRs synthesised at 48 mM CTAB – 12.5 mM NaOL. Other concentrations in the final growth solution were as follows: [$HAuCl_4$] = 482 µM, [$AgNO_3$] = 92.5 µM, [HCl] = 56 mM, [AA] = 620 µM, [$NaBH_4$] = 7.2 µM.

S3.2 48 mM CTAB – 17.5 mM NaOL

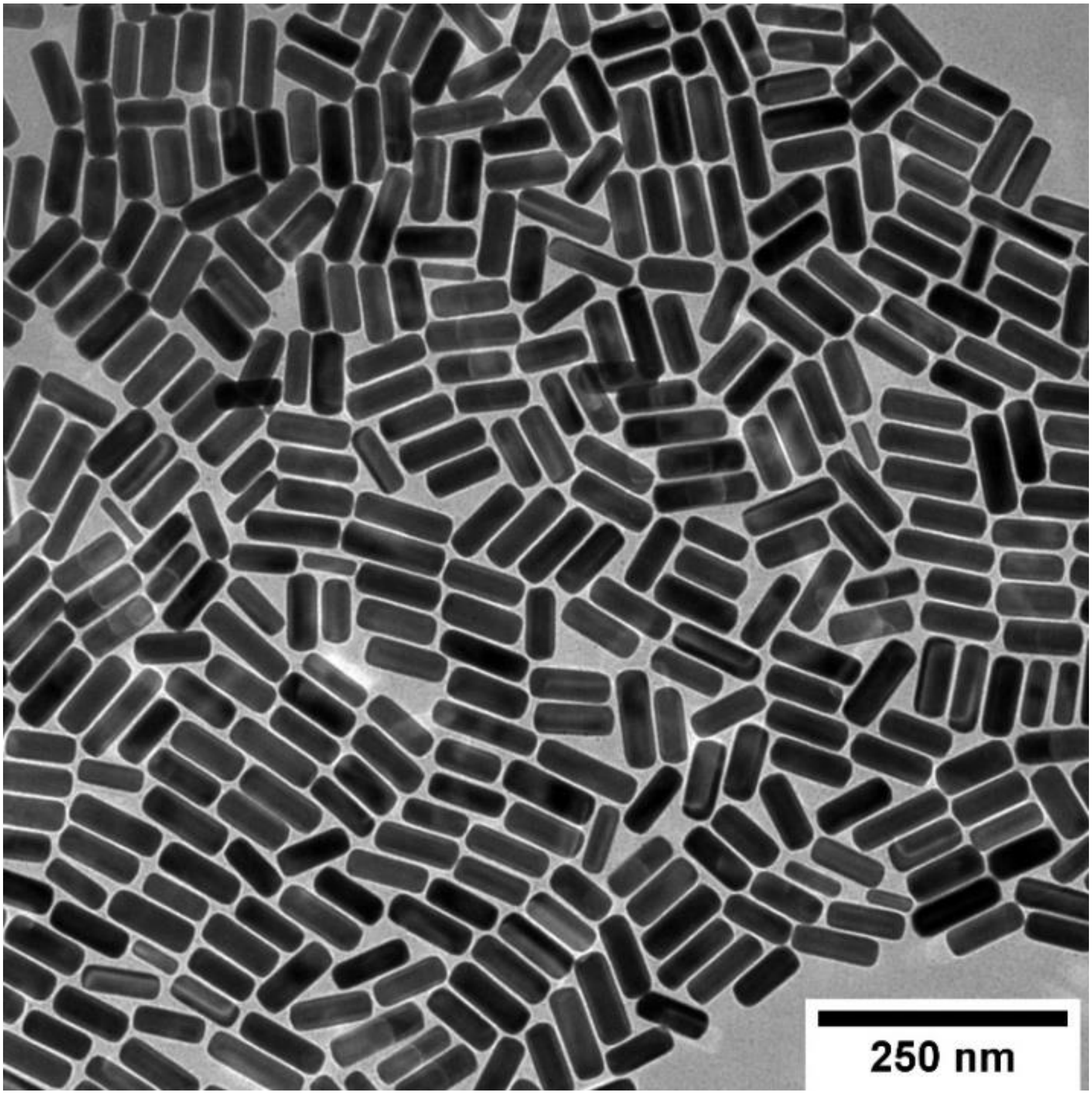

Figure S9. TEM of AuNRs synthesised at 48 mM CTAB – 17.5 mM NaOL. Other concentrations in the final growth solution were as follows: [$HAuCl_4$] = 482 μM, [$AgNO_3$] = 92.5 μM, [HCl] = 56 mM, [AA] = 620 μM, [$NaBH_4$] = 7.2 μM.

S3.3 48 mM CTAB – 20 mM NaOL

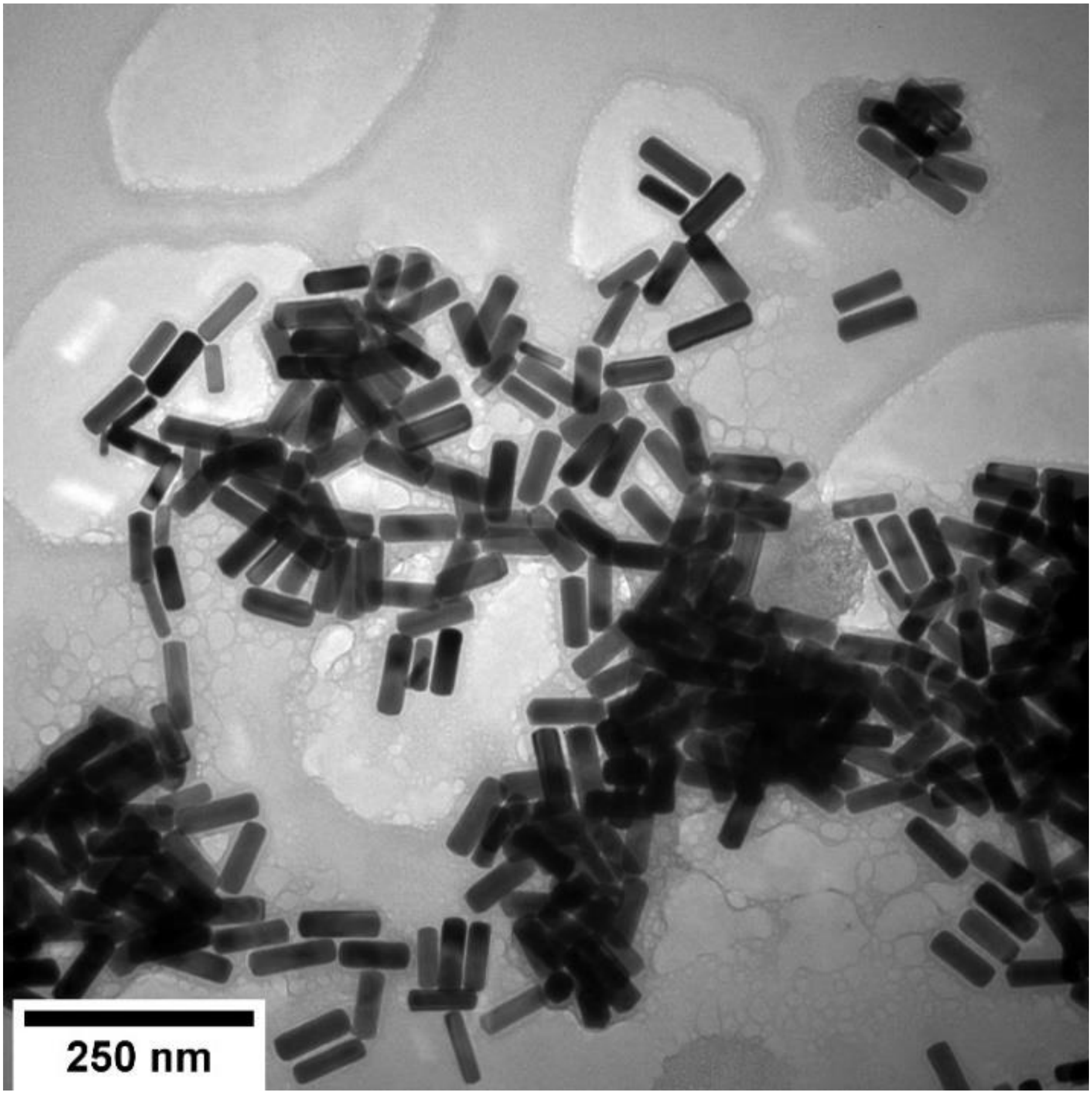

Figure S10. TEM of AuNRs synthesised at 48 mM CTAB – 20 mM NaOL. Other concentrations in the final growth solution were as follows: [$HAuCl_4$] = 482 µM, [$AgNO_3$] = 92.5 µM, [HCl] = 56 mM, [AA] = 620 µM, [$NaBH_4$] = 7.2 µM.

S3.4 48 mM CTAB – 25 mM NaOL

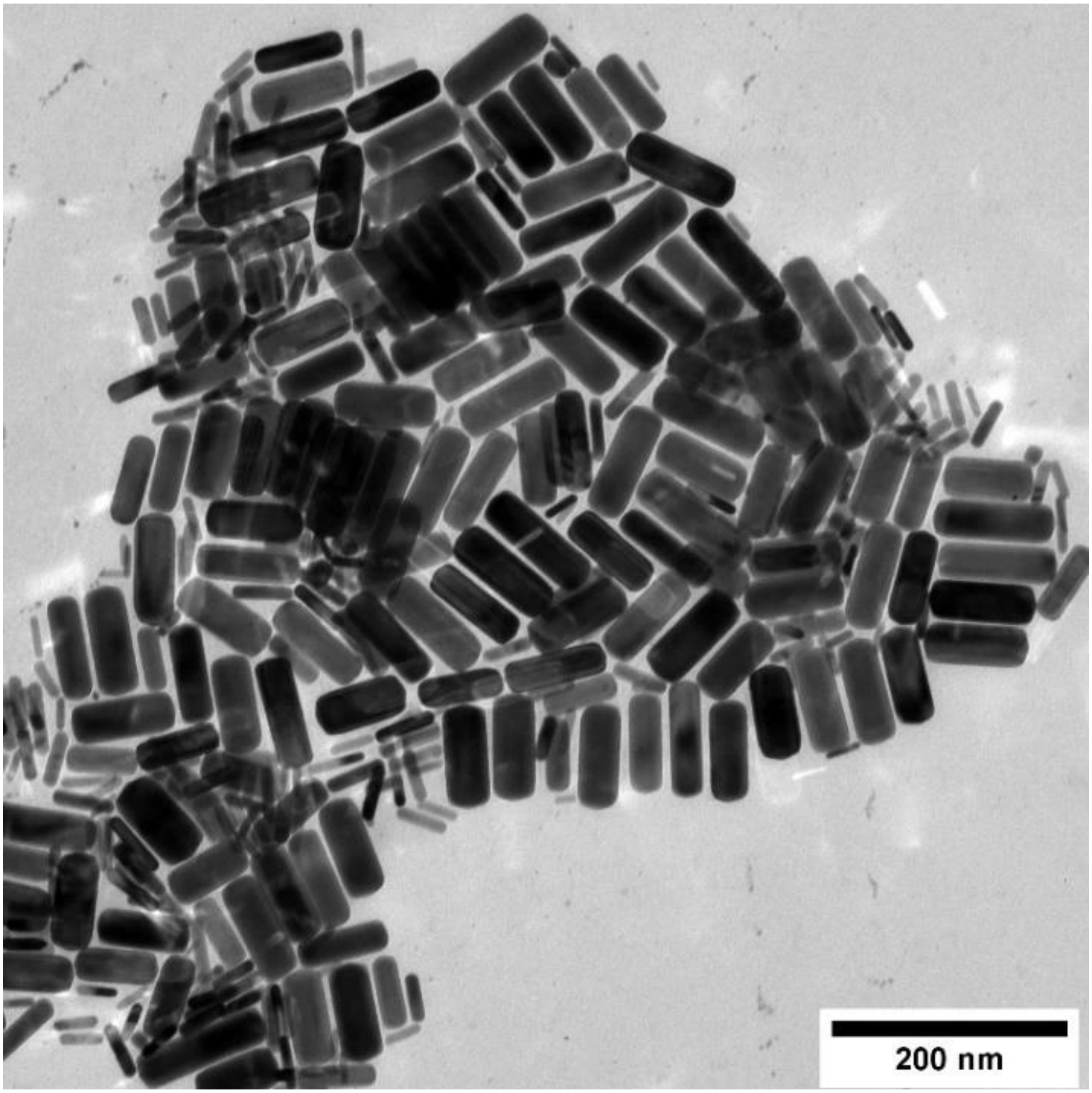

Figure S11. TEM of AuNRs synthesised at 48 mM CTAB – 25 mM NaOL. Other concentrations in the final growth solution were as follows: [$HAuCl_4$] = 482 μM, [$AgNO_3$] = 92.5 μM, [HCl] = 56 mM, [AA] = 620 μM, [$NaBH_4$] = 7.2 μM.
